# Digital Habits and Sleep: Social Media Addiction among University Students

**DOI:** 10.1192/j.eurpsy.2026.11987

**Published:** 2026-07-31

**Authors:** M. A. Said, S. D. Kumaran, M. Ithnin

**Affiliations:** 1 Centre for Epidemiology and Evidence-Based Practice; 2 Department of Social and Preventive Medicine, Universiti Malaya, Kuala Lumpur; 3 School of Health Sciences, KPJ Healthcare University, Nilai, Malaysia

## Abstract

**Introduction:**

Social media is now embedded in the daily lives of university students, shaping patterns of communication, academic involvement, and social interaction. The growing usage of these platforms has generated concerns about digital dependency, with increasing evidence connecting social media addiction to poor sleep quality in Malaysian universities.

**Objectives:**

This study aimed to assess socio-demographic characteristics, patterns of social media use, prevalence of social media addiction, and sleep quality among postgraduate students at Universiti Sains Malaysia (USM), Penang, Malaysia.

**Methods:**

A cross-sectional study was conducted among 402 postgraduate students from various faculties and research centres at USM, Penang. Data were collected using a self-administered questionnaire that included socio-demographic characteristics, the Bergen Social Media Addiction Scale (BSMAS), and the Pittsburgh Sleep Quality Index (PSQI). Descriptive statistics and multivariable logistic regression were applied.

**Results:**

The majority of respondents were female (66.9%), aged >35 years old (52.0%), and enrolled in PhD programmes (59.5%). Most participants accessed social media through smartphones (63.4%), had been using these social media platforms for ≤10 years (72.4%), and reported daily use of ≤4 hours (72.6%). The main purposes for social media use were entertainment (41.5%), knowledge or education (28.4%), and communication (27.1%). More than half of the respondents (54.5%) reported social media addiction, while 58.0% experienced poor sleep quality. Social media use for ≥1 hour before bedtime was associated with 2.37 times higher odds of poor sleep quality, and social media addiction showed a significant association with poor sleep quality (aOR 1.81; 95% CI 1.16–2.83).

**Conclusions:**

The high prevalence of social media addiction and poor sleep quality emphasises the importance of digital health education and behavioural interventions to promote healthier social media usage and improving sleep quality.

**Disclosure of Interest:**

None Declared

